# Insight into immune profile associated with vitiligo onset and anti-tumoral response in melanoma patients receiving anti-PD-1 immunotherapy

**DOI:** 10.3389/fimmu.2023.1197630

**Published:** 2023-08-23

**Authors:** Maria Luigia Carbone, Alessia Capone, Marika Guercio, Sofia Reddel, Domenico Alessandro Silvestris, Daniela Lulli, Carmela Ramondino, Daniele Peluso, Concetta Quintarelli, Elisabetta Volpe, Cristina Maria Failla

**Affiliations:** ^1^ Laboratory of Experimental Immunology, Istituto Dermopatico dell’Immacolata (IDI)-IRCCS, Rome, Italy; ^2^ Laboratory of Molecular Neuroimmunology, Santa Lucia Foundation-IRCCS, Rome, Italy; ^3^ Department of Oncology-Hematology, and Cell and Gene Therapy, Bambino Gesù Children Hospital, IRCCS, Rome, Italy; ^4^ Department of Biology, University “Tor Vergata”, Rome, Italy; ^5^ Department of Clinical Medicine and Surgery, University of Naples Federico II, Naples, Italy

**Keywords:** melanoma, immunotherapy, vitiligo, biomarkers, T-cell receptor

## Abstract

**Introduction:**

Immunotherapy with checkpoint inhibitors is an efficient treatment for metastatic melanoma. Development of vitiligo upon immunotherapy represents a specific immune-related adverse event (irAE) diagnosed in 15% of patients and associated with a positive clinical response. Therefore, a detailed characterization of immune cells during vitiligo onset in melanoma patients would give insight into the immune mechanisms mediating both the irAE and the anti-tumor response.

**Methods:**

To better understand these aspects, we analyzed T cell subsets from peripheral blood of metastatic melanoma patients undergoing treatment with anti-programmed cell death protein (PD)-1 antibodies. To deeply characterize the antitumoral T cell response concomitant to vitiligo onset, we analyzed T cell content in skin biopsies collected from melanoma patients who developed vitiligo. Moreover, to further characterize T cells in vitiligo skin lesion of melanoma patients, we sequenced T cell receptor (TCR) of cells derived from biopsies of vitiligo and primary melanoma of the same patient.

**Results and discussion:**

Stratification of patients for developing or not developing vitiligo during anti-PD-1 therapy revealed an association between blood reduction of CD8-mucosal associated invariant T (MAIT), T helper (h) 17, natural killer (NK) CD56^bright^, and T regulatory (T-reg) cells and vitiligo onset. Consistently with the observed blood reduction of Th17 cells in melanoma patients developing vitiligo during immunotherapy, we found high amount of IL-17A expressing cells in the vitiligo skin biopsy, suggesting a possible migration of Th17 cells from the blood into the autoimmune lesion. Interestingly, except for a few cases, we found different TCR sequences between vitiligo and primary melanoma lesions. In contrast, shared TCR sequences were identified between vitiligo and metastatic tissues of the same patient. These data indicate that T cell response against normal melanocytes, which is involved in vitiligo onset, is not typically mediated by reactivation of specific T cell clones infiltrating primary melanoma but may be elicited by T cell clones targeting metastatic tissues. Altogether, our data indicate that anti-PD-1 therapy induces a de novo immune response, stimulated by the presence of metastatic cells, and composed of different T cell subtypes, which may trigger the development of vitiligo and the response against metastatic tumor.

## 1 Introduction

Melanoma is an aggressive skin cancer, whose incidence rates have increased over the past few decades not only in adults, but also in children and adolescents. Cutaneous melanoma is characterized by a high mutational burden, providing a consistent number of antigens that could constitute targets of the immune response (1). Indeed, melanoma-specific cytotoxic T lymphocytes have been observed in patient’s blood and melanoma tissues (2). However, this immune response rarely succeeds in effective tumor clearance. It is evident that tumor progression must be accompanied by impairment of immune responses and to overcome such an impairment, therapeutic treatments based on immune checkpoint inhibitors have been developed and proved highly effective in cutaneous melanoma (3). Nevertheless, the overall response rate observed in unresectable/metastatic stage III-IV melanoma patients treated with anti-programmed death (PD)-1 antibodies as monotherapy, is still low, around 40% (4). Thus, availability of early markers of response to treatment would permit to interrupt the therapy for not-responsive patients, promptly switching them towards alternative therapeutic approaches, and reducing the risk of developing immune-related adverse events (irAE). Among the possible irAE emerging during anti-PD-1 treatment, melanoma patients develop leukoderma lesions, also called vitiligo lesions due to their high similarity to those of the spontaneous autoimmune skin disease vitiligo. In fact, histopathological aspects of immunotherapy-induced vitiligo were almost indistinguishable from spontaneous vitiligo (5). Of note, development of vitiligo seems to be specific of cutaneous melanoma, as it is rarely reported as an irAE during other tumor type treatments (5); antibodies and CD8^+^ T lymphocytes directed against melanocyte-specific antigens have been identified in both cases, suggesting that, in cutaneous melanoma, vitiligo appearance could reflect a broad immune cell activation, also effective against cancer cells (6). Actually, we and others have demonstrated that the onset of vitiligo during treatment with checkpoint inhibitors correlates with a better outcome in metastatic melanoma patients (6–9). Nevertheless, the mechanism underlying the association between vitiligo development and tumor regression is still unclear. Previously, it has been shown that the same T lymphocyte clone was present in a primary melanoma and in a vitiligo lesion spontaneously developed in the same patient (10). This result suggested that vitiligo appearance was exerted by T cells directed against antigens shared by primary melanoma and normal melanocytes. More recently, a patient affected by widespread uveal melanoma and treated with anti-cytotoxic T-lymphocyte antigen (CTLA)-4/anti-PD-1 therapy showed an exceptional response accompanied by the development of several irAEs, comprising vitiligo. TCR-sequencing of the primary tumor, as well as of different metastatic samples, identified identical T cell clones in the examined tissues (11). Certainly, future researchers would benefit from a deeper understanding of this immunological mechanism, also in order to determine the best application of vaccine-based or T cell-based therapies, currently under study in clinical trials for the treatment of cutaneous melanoma.

Similarities in immune molecular processes involved in autoimmunity and in anti-tumor responses have already been observed (12). Indeed, cancer-specific mortality was significantly lower in patients with an autoimmune background (12), and accordingly cancer patients showing clinically relevant anti-tumor immune responses accompany with a “beneficial autoimmunity”, also important in the processes of cancer immunosurveillance (13). In this context, we have hypothesized that the immunological molecular pathways observed in vitiligo may also occur in melanoma patients who show positive response to immunotherapy. In a previous study, we demonstrated that soluble CD25 (sCD25) and CXCL9, two known circulating vitiligo-specific biomarkers, were also higher in sera of stable and responsive metastatic melanoma patients undergoing anti-PD-1 therapy compared to non-responders (14). Moreover, peripheral blood mononuclear cells (PBMCs) of responders showed a lower blood frequency of T regulatory lymphocytes (T-reg), a suppressive T cell immune population, compared to non-responder patients, either before or after three months of anti-PD-1 therapy (14). Altogether, these data underlined the presence of immune mechanisms in cutaneous melanoma, common to spontaneous vitiligo and potentially mediating both autoimmune responses accountable for vitiligo development and anti-tumor immune responses. As a further demonstration of this hypothesis, in the present study we extended the analysis to circulating- and tissue-related immune cells, comparing frequency and activation features in patients who developed or not vitiligo under anti-PD-1 therapy. Our results give insight into the immune mechanisms which mediate the onset of this irAE and that may predict the occurrence of an effective anti-tumor immune response.

## 2 Materials and methods

### 2.1 Patients

This study was conducted according to the Good Clinical Practice Guidelines and the Declaration of Helsinki. The study was approved by the Institutional Review Boards of Istituto Dermopatico dell’Immacolata (IDI)-IRCCS (510/3, April 2018). All patients enrolled in the study provided written informed consent. This study included patients with unresectable metastatic melanoma, stage IIIc or IV based on American Joint Committee on Cancer (AJCC, version 7) staging (15), enrolled for treatment with anti-PD-1 inhibitors at IDI-IRCCS. Peripheral blood samples were collected before therapy and at every therapy administration up to one-year, disease progression or vitiligo appearance. Nivolumab (Opdivo^®^) was given at the dose of 480 mg every 4 weeks, pembrolizumab (Keytruda^®^) at the dose of 200 mg every 3 weeks. Patients underwent physical examination and assessment of biochemical parameters monthly, whereas investigator-determined objective response was assessed radiologically with computed tomography scans approximately every 12 weeks after treatment initiation. Tumor response was classified as immune complete response (iCR), partial response (iPR), or stable disease (iSD), according to the immune response evaluation criteria in solid tumors (iRECIST 1.1) (16, 17). Therapy efficacy evaluation was based on best overall response (iOR) determined as best time point response according to iRECIST.

### 2.2 PBMC isolation and cytofluorimetric analysis

Whole blood samples were collected into vacutainer sodium citrate tubes (cat. no. 367704, BD Biosciences, Plymouth, UK) and PBMCs were isolated by Ficoll gradient centrifugation (GE Healthcare, Little Chalfont, UK). Cryopreserved PBMCs were stained with the following antibodies, as previously described (14): Panel 1- anti-human CD4 FITC (1:100) (cat. no. 130-114-531, Miltenyi Biotech, Auburn, CA, USA) (1:100), anti-human CRTh2-PE (1:150) (cat. no. 130-114-128, Miltenyi Biotech), anti-human CD161-PE/Dazzle594 (1:50) (cat. no. 339939, Biolegend, San Diego, CA, USA), anti-human CD3 PercP-Cy5.5 (1:300) (cat. no. 300327, Beckman Coulter, Brea, CA, USA), anti-human CXCR3-APC Alexa647 (1:40) (cat. no. 353711, Biolegend), anti-human CD8-APC Alexa700 (1:120) (cat. no. A66332, Beckman Coulter), anti-human CCR6-BV421 (1:30) (cat. no. 353407, Biolegend), anti-human PD1-BV650 (1:30) (cat. no. 564104, BD Biosciences), and LIVE/DEAD™ Fixable Aqua Dead Cell Stain Kit (1:200) (cat. no. l34957, Invitrogen, Waltham, MA, USA). Panel 2: anti-human CD4 FITC (1:100) (cat. no. 130-114-531, Miltenyi Biotech), anti-human CD3-ECD (1:100) (cat. no. IM2705U, Beckman Coulter), anti-human CD127-APC Alexa700 (1:200) (cat. no. 351343, Beckman Coulter), anti-human CD25-BV421 (1:60) (cat. no. 564033, BD Biosciences), anti-human PD1-BV650 (1:30) (cat. no. 564104, BD Biosciences), and LIVE/DEAD™ Fixable Aqua Dead Cell Stain Kit (1:200) (cat. no. l34957, Invitrogen). Samples were acquired using Cytoflex cytometer (Beckman Coulter) and analyzed using FlowJo-10 software version 10.3.0. Gating strategy for discrimination of different cell populations is described in **Supplementary Figure 1**. Briefly, CD3 (CD3^+^); CD4 (CD3^+^, CD4^+^); CD8 (CD3^+^, CD8^+^); CD8-MAIT (CD3^+^, CD8^+^, CD161^high^) as previously reported (18, 19); NK cells (CD3^-^, CD56^dim^); NK bright cells (CD3^-^, CD56^high^); TCR-γδ (CD3^+^, TCR-γδ^+^); B cells (CD3^-^, CD19^+^); naïve CD4 T cells (CD3^+^, CD4^+^, CD45RA^high^), memory CD4 T cells (CD3^+^, CD4^+^, CD45RA^-^). For CD4 T cells we performed analysis as previously reported (20, 21): Th1 (CD3^+^, CD4^+^, CRTH2^-^, CXCR3^+^, CCR6^-^); Th17 (CD3^+^, CD4^+^, CRTH2^-^, CXCR3^-^, CD161^+^, CCR6^+^); Th1/17 (CD3^+^, CD4^+^, CRTH2^-^, CXCR3^+^, CD161^+^, CCR6^+^); Treg (CD3^+^, CD4^+^, CD127^-^, CD25^high^). All cell populations were analyzed within alive cells, excluding debris and doublets.

### 2.3 Immunohistochemistry and immunofluorescence

Formalin-fixed, paraffin-embedded (FFPE) sample blocks of normal skin (2 patients), primary melanomas (10 patients), metastases (5 samples from 3 patients), or vitiligo in patients without concomitant melanoma (10 patients), were collected from the archive of the IDI-IRCCS Histopathology Unit. Biopsies from melanoma patients who developed vitiligo during therapy (10 patients) were surgically taken at the lesion margin, fixed in 10% formalin, and embedded in paraffin. Four-µm sections were obtained from each sample, dewaxed, and rehydrated. After quenching endogenous peroxidase, achieving antigen retrieval, and blocking non-specific binding sites, sections were incubated with the following antibodies: mouse monoclonal antibody anti-CD25 (cod. LSBio-B7396-50; LS Bio, Seattle, WA; 1:5); mouse monoclonal antibody anti-interleukin (IL)-17A (cod. AF-317-NA, R&D Systems, Minneapolis, MSP; 1:30); monoclonal antibody anti-CD56 (cod. NCL-CD56, Novocastra Scytek, Wetzlar, Germany; 1:20), mouse monoclonal antibody anti-CD3 (cod. A0452, Dako, Santa Clara, CA; 1:100), mouse monoclonal antibody anti-CD8 (cod. CM154A, Biocare Medical, Concord, CA; 1:75) overnight at 4°C in a humid chamber. Secondary biotinylated polyclonal Abs and staining kits were obtained from Vector Laboratories (Burlingame, CA). Immunoreactivity was visualized with peroxidase reaction using 3-amino-9-ethylcarbazole (AEC) in H_2_O_2_ and specimen counterstained with hematoxylin. As a negative control, primary Abs was omitted. Stained sections were analyzed with the AxioCam digital camera coupled to the Axioplan 2 microscope (Carl Zeiss AG, Oberkochen, Germany). Staining intensity was evaluated by quantitative analysis (Image J color deconvolution) in five adjacent fields of each section by two independent observers, blinded to the status of the specimens. For immunofluorescence, 4-µm sections were dewaxed, rehydrated, and after quenching endogenous peroxidase with 3% bovine serum albumin in 1x phosphate buffer, achieving antigen retrieval and blocking non-specific binding sites, sections were incubated with the monoclonal antibody anti-TCR V alpha 7.2 FITC conjugated (cod.130-123-929, Miltenyi Biotech; 1:50) and incubated for 1 hour at 37°C in a humid chamber. After a few washes, sections were closed using mounting medium with DAPI for stained nuclei (Antifade Mounting Medium, Vectashield, Vector Laboratories). Images were acquired with the ApoTome System (Zeiss) connected with an Axiovert200 inverted microscope (Zeiss); image analysis was performed with ZEN software (Zeiss). Fluorescence was evaluated by quantitative analysis (Image J color deconvolution) in three adjacent fields of each section by two independent observers, blinded to the status of the specimens.

### 2.4 TCR sequencing

*Sample preparation.* Five to 10 slices of 5 µm thickness from vitiligo or primary/metastatic melanoma biopsies from the same patient were obtained from FFPE samples and used for DNA extraction. For tissue samples, QIAamp DNA FFPE Tissue Kit (Qiagen, Valencia, CA) was used in conjunction with an incubation at 55°C for 4 hours to complete tissue lysis. Next, samples were incubated at 90°C for 1 hour to reverse formaldehyde modification of nucleic acids. For blood samples, DNA extraction from PBMCs was performed following the DNeasy Blood & Tissue Kit (Qiagen). Proteinase K was used for digestion and extraction of DNA following a blood/cell protocol with RNase treatment and using spin-column method. After isolation by QIAamp MinElute column (Qiagen), variable amounts of buffer were added to each column to elute the DNA. Samples were quantified using Dropsense96 and diluted for library preparation in DEPC water.

*Library preparation.* Extracted DNA was used for TCR Vβ analysis using the ImmunoSEQ hsTCRB Kit (Adaptive Biotechnologies, Seattle, WA), according to the manufacturer’s instructions. For PBMCs and metastasis samples, 1 µg of total DNA was used for duplicate, whilst for primary melanoma samples, 200-1000 ng of total DNA was used for duplicates. For FFPE vitiligo samples, the staring gDNA was poor (50-325 ng) and, when possible, sequencing was repeated in order to increase the output. Due to the *post-hoc* nature of this study, we decided to perform the sequencing as well, at the best of our conditions using at least 50 ng of starting gDNA. The somatically rearranged human CDR3 was amplified from genomic DNA using a two-step, amplification bias-controlled multiplex PCR approach (22–24). The first PCR consists of forward and reverse amplification primers specific for every V and J gene segment and amplifies the hypervariable complementarity-determining region 3 (CDR3) of the immune receptor locus. The second PCR adds a proprietary barcode sequence and Illumina^®^ adapter sequences (25, 26). Following purification with the Agencourt™ AMPure™ XP Reagent (Beckman Coulter, Inc.), 10 µl of each sample were pooled in equimolar concentration into three final libraries (46 libraries/pool). Final libraries were quantified using KAPA Library Quantification Kits (Roche, Switzerland, CH), diluted to 4 nM, and denatured using 0.2N NaOH.

*Sequencing.* CDR3 libraries were loaded at 20 pM on an Illumina NextSeq 550 system with 156 paired-end sequencing (Illumina, San Diego, CA). Sample data was generated using the immunoSEQ^®^ Assay (Adaptive Biotechnologies, Seattle, WA). For the runs, ~146.92M reads where generated and ~128.58M passing filter. The coverage for each sample varied with the highest being 92x. The release of sequencing data and the QC control were performed by Adaptive Biotechnologies technical support.

The starting material (gDNA) was not always compliant to manufacturer’s instructions. When possible, the sequencing was repeated using other specimens, to increase the output. As this was a small cohort, the threshold we established is considered exploratory.

*Data Analysis.* Raw sequence reads were demultiplexed according to Adaptive’ s proprietary barcode sequences. Demultiplexed reads were then further processed to remove adapter and primer sequences; identify and correct for technical errors introduced through PCR and sequencing; and remove primer dimer, germline, and other contaminant sequences. Data were filtered and clustered using both the relative frequency ratio between similar clones and a modified nearest-neighbor algorithm, to merge closely related sequences. The resulting sequences were sufficient to allow annotation of the V(N)D(N)J genes constituting each unique CDR3 and the translation of the encoded CDR3 amino acid sequence. V, D and J gene definitions were based on annotation in accordance with the IMGT database (www.imgt.org). The set of observed biological human CDR3 sequences were normalized to correct for residual multiplex PCR amplification bias and quantified against a set of synthetic human CDR3 sequence analogues (26, 27). Data was analyzed using the immunoSEQ Analyzer toolset (ImmunoSEQ Analyzer 3.0 https://clients.adaptivebiotech.com/). Libraries were sequenced and organized providing productive and non-productive sequences (CDR3 regions explicitly encoding a premature stop, and those predicted to put the receptor gene out-of-frame downstream of the CDR3 rearrangement, were considered non-productive). Productive TCR-β CDR3 frequencies were used for generating scatterplots. Additional analyses were performed using GraphPad prism (GraphPad Software) and the R graphical library ggplot2. Alluvial flow diagrams (28) were used to describe common TCR sequences that can be viewed as multiple streams that flow smoothly throughout different samples of the same patient (primary melanoma, vitiligo and, if present, metastasis). To generate an alluvial map, common V(D)J rearrangements data from ImmunoSeq Analyzer were used in order to generate a series of networks for shared TCR sequence across different specimens, and these networks were loaded into the alluvial generator (gg alluvial library; https://cran.r-project.org/web/packages/ggalluvial/vignettes/ggalluvial.html).

Details on V(D)J usage for each productive sequence in the samples are reported as **VDJ usage** **Supplementary File**. For each patient, the nucleotide sequence of CDR3 unique region of shared TCR-β clonotypes and the productive frequency are listed in **Supplementary Table II**.

### 2.5 Statistical analysis

For TCR Vβ statistical analysis, a limited number of findings were evaluated for statistical significance. Groups were compared using the Producting Simpson Clonality Index (ImmunoSEQ™ software) and Kruskal-Wallis nonparametric test was used to analyze the difference in TCR clonality between different sample types. Two-stage linear step-up procedure of Benjamini, Krieger and Yekutieli was used for *post-hoc* analysis.

For flow cytometry data, differences between pairs were analyzed by paired Student’s t-test, and multiple comparisons by two-way ANOVA test.

For tissue analyses, before proceeding with the hypothesis tests, we analyzed the data of each group through a Shapiro-Wilk test which highlighted a non-normal distribution of our observations. Furthermore, given some values ​​far from the mean for some groups and the limited data available, we decided to compare the experimental groups using a Kruskal-Wallis test which is a non-parametric test which highlights the differences between the medians. Statistical significance was set at p<0.05 and all statistical analyzes were conducted using GraphPad Prism software (La Jolla, CA, USA) and R.

## 3 Results

### 3.1 Patients who developed vitiligo during immunotherapy have a diverse frequency of circulating immune cells

**Figure 1:**
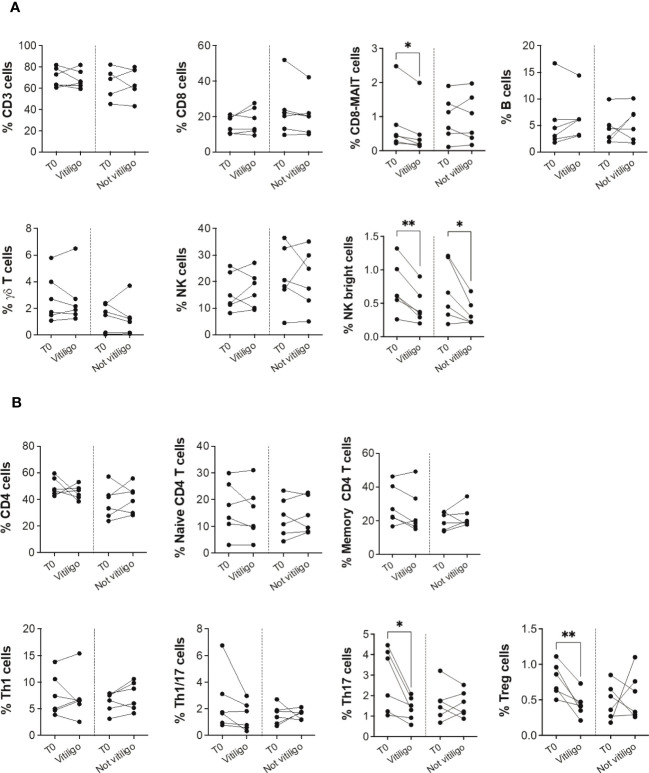
Vitiligo development due to anti-PD-1 immunotherapy alters the frequencies of subsets of circulating immune cells. **(A)** Blood frequencies of CD3^+^, CD8^+^, CD8-MAIT, B, γδ T, NK, NK bright cells and **(B)** several CD4^+^ cell subsets were analyzed by flow cytometry in PBMCs from melanoma patients before therapy (T0), at the time of vitiligo onset during therapy (Vitiligo), or at a corresponding control time for patients who did not develop vitiligo (Not vitiligo). Frequencies of each cell subset are reported as percentage within parental gate, which is defined in bold above each dot plot of **Supplementary Figure 1**. Data are indicated as mean value ± standard error of the mean (SEM). *p<0.05, **p<0.005 as assessed by paired t-test or two-way ANOVA test.

To investigate whether the immune profile of melanoma patients undergoing anti-PD-1 immunotherapy was associated to the irAE vitiligo, we performed flow cytometry analyses on patients’ PBMCs. Twelve patients who had a positive response to anti-PD-1 immunotherapy were examined: six developed vitiligo during therapy, while the other six did not. After stratification of patients into two groups matched for sex and age, we compared immune cell frequency considering as the unique variant the development of vitiligo (**Table 1**). Cells were isolated from blood samples taken before therapy initiation (T0), and on-treatment at each administration up to one year or at vitiligo development. For the comparison, two time points of collection were considered: before therapy (T0) and at the number of treatment when vitiligo onset was observed, for patients who developed vitiligo; before therapy (T0) and at treatment number 4 or 5, for patients who did not develop vitiligo. Control time points were chosen according to the onset of vitiligo, which did not occur earlier than the fourth therapeutic administration in the matching group. Our results showed a reduction of circulating CD8-mucosal associated invariant T (MAIT), T helper (Th)-17, and T-reg cells associated to vitiligo onset. Moreover, a reduction in natural killer (NK) CD56^bright^ cell frequency was detected during treatment in both patient groups, suggesting its association with a positive clinical response (**Figures 1A, B** **)**.

**Table 1:** Characteristics and treatment outcome of melanoma patients who developed or not vitiligo during therapy.

	Patients who developed vitiligo									Patients who did not develop vitiligo							
	Stage^a^	Checkpoint inhibitor^b^	BRAF	Sex^c^	Age^d^	iOR	PBMCs			Stage^a^	Checkpoint inhibitor^b^	BRAF	Sex^c^	Age^d^	iOR	PBMCs	
							Before therapy	Vitiligo onset								Before therapy	Control time
IMM-1	M1b	Pembro	MUT	M	72	iCR	T0	T10	IMM-7	M1a	Pembro	WT	M	89	iPR	T0	T5
IMM-2	M1d	Pembro	WT	M	84	iSD	T0	T4	IMM-8	M1a	Pembro	WT	M	68	iPR	T0	T5
IMM-3	M1a	Pembro	WT	F	91	iPR	T0	T6	IMM-9	M1c	Nivo	WT	M	86	iCR	T0	T4
IMM-4	M1c	Nivo	NA	M	80	iPR	T0	T12	IMM-10	M1c	Nivo	MUT	M	71	iPR	T0	T4
IMM-5	IIIc	Nivo	WT	M	55	iCR	T0	T10	IMM-11	M1b	Nivo	WT	F	69	iCR	T0	T4
IMM-6	M1b	Pembro	WT	M	81	iPR	T0	T8	IMM-12	M1b	Nivo	WT	F	53	iPR	T0	T5

^a^Staging before starting therapy; ^b^Pembro, pembrolizumab; Nivo, nivolumab; ^c^M, male; F, female; ^d^Age in years at the time of therapy initiation; iOR: Immune Objective Response; iCR, immune complete response, iSD, immune stable disease, iPR, immune partial response. BRAF: analysis for BRAF600E mutation. MUT= presence of the mutated gene; WT, wild type; NA, not available. PBMCs, peripheral blood mononuclear cells. Blood samples were taken before therapy initiation (T0). The number of therapeutic treatments (T) that patients underwent when the onset of vitiligo occurred, corresponds to the time in which PBMCs were isolated. For patients who did not develop vitiligo, a corresponding therapeutic treatment point (T) was chosen as a control.

**Figure 2:**
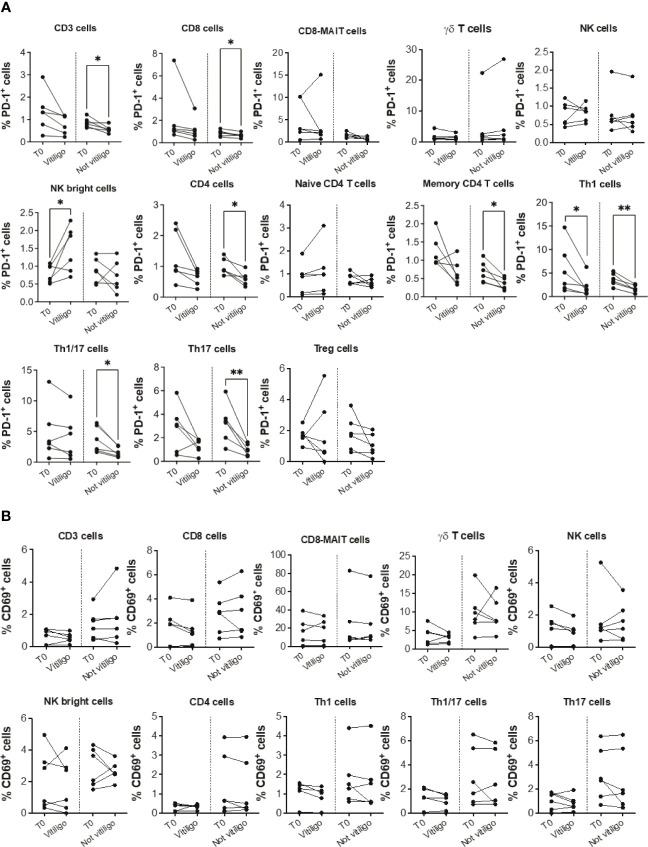
Vitiligo development during anti-PD-1 therapy alters PD-1 and CD69 expression in circulating immune cells. Percentage of PD-1 positive cells **(A)** and CD69 positive cells **(B)** gated within CD3^+^, CD8^+^, CD8-MAIT, γδ T, NK, NK bright cells and within several CD4+ cell subsets was analyzed by flow cytometry in PBMCs from melanoma patients before therapy (T0), at the time of vitiligo onset during therapy (Vitiligo), or at a corresponding control time for patients who did not develop vitiligo (Not vitiligo). Data are indicated as mean value ± standard error of the mean (SEM). *p<0.05, **p<0.005 as assessed by paired t-test or two-way ANOVA test.

To analyze immune cell subtypes potentially responding to anti-PD-1 immunotherapy, we examined PD-1 expression in different immune cell subsets. A reduction in the frequency of PD-1 expressing CD3^+^, CD4^+^, CD8^+^, Th1, Th1/17, and Th17 cells was seen in patients not experiencing vitiligo during therapy (**Figure 2A**). A trend of such a reduction was also observed in patients developing vitiligo but was significant only for Th1 cells. Conversely, as therapy cycles increase, increased PD-1 expression of NK CD56^bright^ cells distinguished patients developing vitiligo (**Figure 2A**). In order to evaluate the activation status of different immune cell subtypes, we examined the expression of CD69, classical early marker of lymphocyte activation due to its rapid appearance on the surface of the plasma membrane after stimulation of different immune cell subsets (29). As shown in **Figure 2B**, there were neither significant changes in CD69 expression during therapy nor differences in CD69 expression before therapy in patients who developed vitiligo or not. **Supplementary Figures 2, 3** report the frequency data obtained for each patient at every time-point of treatment.

### 3.2 Differential immune profile in immunotherapy-induced and spontaneous vitiligo

**Figure 3:**
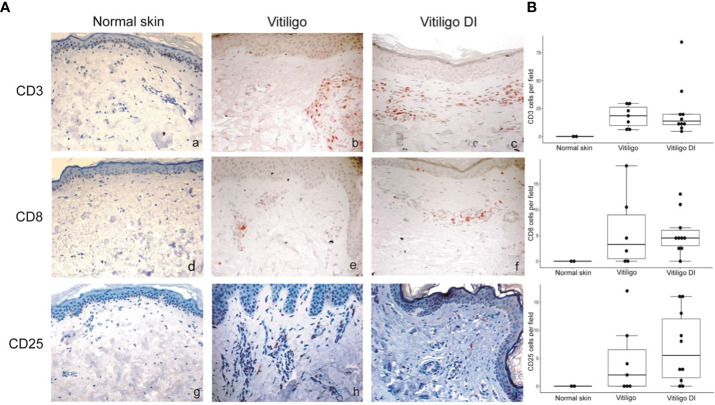
Vitiligo tissue expression of the immune cell subsets modulated differently in the circulation of melanoma patients developing vitiligo during anti-PD-1 therapy. **(A)** Representative images of immunohistochemistry analysis performed using antibodies directed against CD3 (panels a–c), CD8 (panels d–f), and CD25 (panels g–i), on normal skin biopsies and on biopsies from patients affected by vitiligo without melanoma (vitiligo) and who developed vitiligo during immunotherapy (vitiligo DI). Magnification 200x. **(B)** Quantitative analyses of immunohistochemical staining. Data are indicated as mean value + standard error of the mean (SEM), as assessed by Kruskal-Wallis test.

**Figure 4:**
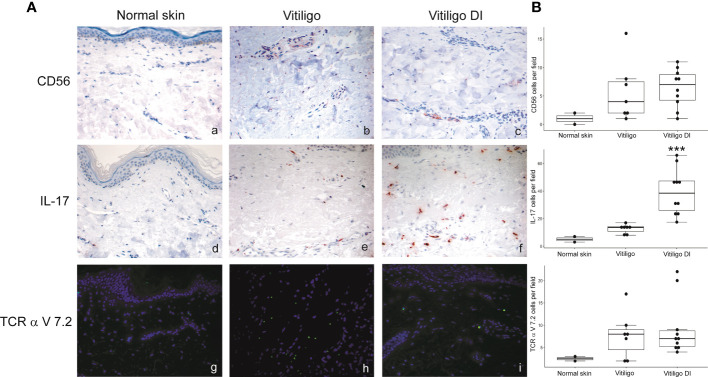
IL-17-expressing cells are differently modulated in the skin lesion and in the circulation of melanoma patients developing vitiligo during anti-PD-1 therapy. **(A)** Representative images of the immunohistochemistry (panels a to d) and immunofluorescence (panels e, f) analysis performed using antibodies directed against CD56 (panels a–c), IL-17 (panels d–f) and TCR alpha V 7.2 (panels g–i) on normal skin biopsies and on biopsies from patients affected by vitiligo without melanoma (vitiligo) and who developed vitiligo during immunotherapy (vitiligo DI). Magnification 200x. **(B)** Quantitative analyses of immunohistochemical staining. The mean value + standard error of the mean (SEM) of the cell count obtained for five different fields is shown, ***p<0.05 as assessed by Kruskal-Wallis test.

To analyze at the tissue level the role of immune cells differently modulated in melanoma patients developing vitiligo, we performed immunohistochemistry and immunofluorescence analyses on vitiligo specimens collected from ten melanoma patients treated with anti-PD-1 antibodies (**Table 2**). We compared them with the specimens from ten patients developing vitiligo in the absence of concomitant melanoma or immunotherapy. Two normal skin specimens were used as a control. As shown in **Figures 3**, **4**, we observed a trend of increased number of cells positive to an anti-CD25 antibody, that could correspond to T-reg cells or other activated conventional T cells, and a significant increment of cells stained with an anti-IL-17A antibody in vitiligo lesions induced by immunotherapy compared to the other vitiligo samples. Interestingly, this high number of IL-17-positive cells detected in immunotherapy-induced vitiligo lesions may suggest the recruitment of Th17 cells *in situ*, according to the decrease of circulating Th17 cells observed over treatment in this patient population (**Figure 1**). Although not significant, we observed a lower amount of TCR V alpha 7.2 positive cells, possibly MAIT cells, in the immunotherapy-derived vitiligo patients compared to vitiligo samples without melanoma.

**Table 2:** Characteristics and TCR clonotype number of melanoma patients who developed vitiligo during therapy.

	Stage^a^	Checkpoint inhibitor^b^	BRAF	Sex^c^	Age^d^	iOR	TCR clones shared by primary melanoma and vitiligo (N)	TCR clones shared by primary melanoma and PBMCs (N)	TCR clones shared by vitiligo and PBMCs (N)	TCR clones shared by metastasis and vitiligo (N)	TCR clones shared by metastasis and PBMCs (N)	TCR clones shared by metastasis and vitiligo (N)
VIT-1	M1b	Pembro	MUT	M	69	iCR	22	9	11	–	–	–
VIT-2	M1a	Pembro	WT	F	48	iPR	1	317	9	5	241	5
VIT-3	M1a	Nivo	WT	F	72	iPR	1	5	6	–	–	–
VIT-4	M1c	Nivo	WT	F	54	iPR	2	3	0	12	6	12
VIT-5	M1d	Nivo	MUT	F	41	iPR	8	68	29	14	307	14
VIT-6	M1b	Pembro	WT	F	53	iPR	ND	1	3	–	–	–
VIT-7	M1a	Nivo	WT	M	56	iPR	4	19	23	–	–	–
VIT-8	M1a	Nivo	WT	M	66	iPR	2	6	12	9 (spleen) and 32 (lymph node)	28 (spleen) and 112 (lymph node)	9 (spleen) and 32 (lymph node)
VIT-9	M1b	Nivo	WT	F	73	iPR	ND	7	5	–	–	–
VIT-10	M1c	Pembro	WT	M	83	iCR	13	50	18	–	–	–

^a^Staging before starting therapy; ^b^Pembro, pembrolizumab; Nivo, nivolumab. BRAF: analysis for BRAF600E mutation. MUT= presence of the mutated gene; WT,wild type; ^c^M, male; F, female; ^d^Age in years at the time of therapy initiation; iOR: Immune Objective Response; iCR, immune complete response, iPR, immune partial response; ND, not detected shared clones; PBMC samples were sequenced at the time of vitiligo onset.

### 3.3 T-cells infiltrating immunotherapy-induced vitiligo share V(D)J rearrangements with T-cells infiltrating metastasis lesions and primary melanomas

To further characterize T cells in vitiligo lesions of melanoma patients, we performed sequencing of CDR3 variable region of the β-chain of the T-cell receptor (TCR-seq) on gDNA extracted from samples of vitiligo biopsies. Moreover, from the same patient, we have also carried out a TCR sequencing of PBMCs at the time of vitiligo onset, and on retrospective samples from the same patient of primary melanoma and of metastasis sites, when occurred (n=4). **Table 2** summarizes the melanoma patients included in the TCR-Seq, and their clinical features, whereas **Supplementary Table I** reports the most relevant metrics from the TCR-Seq for all the analyzed samples, including total and productive templates and rearrangements, as well as the productive and sample Simpson clonality of the TCR-seq.

**Figure 5:**
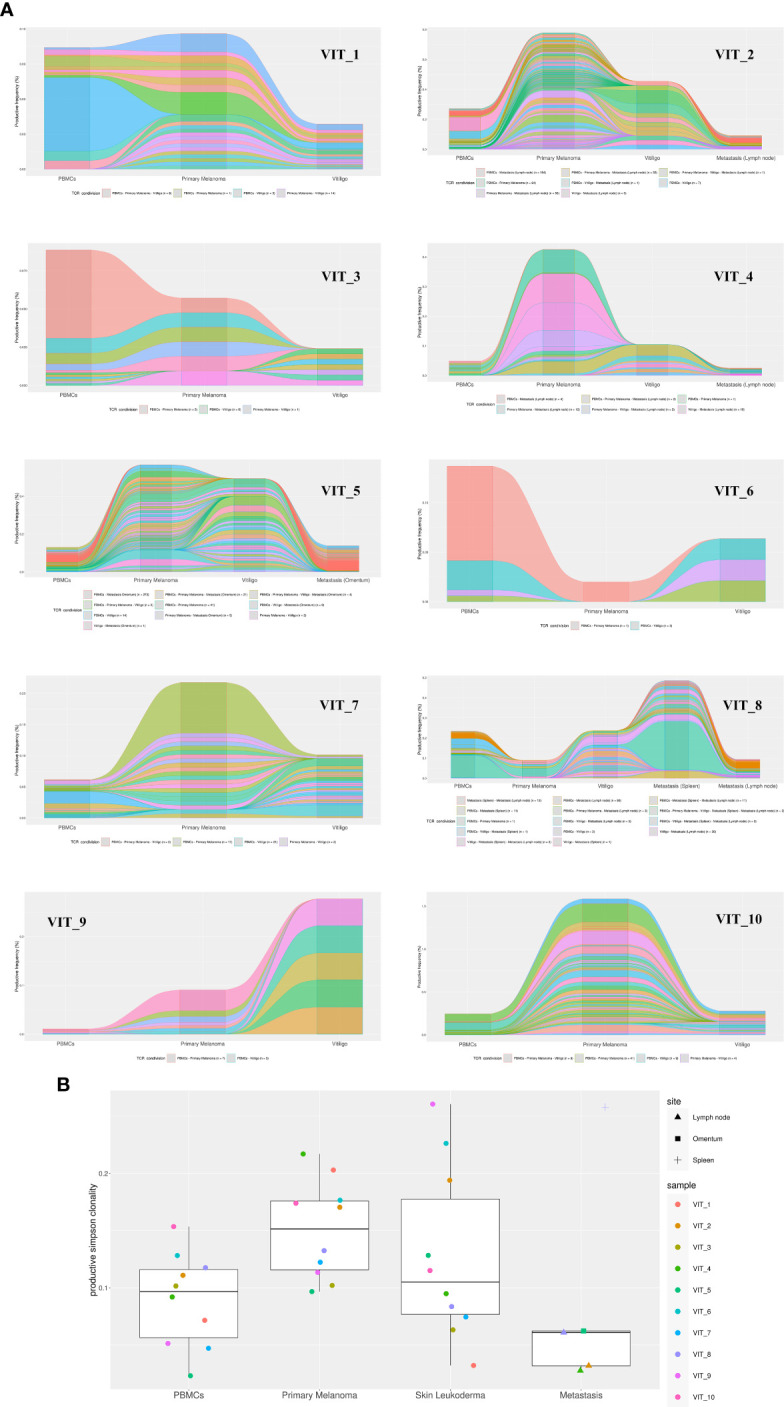
TCR-Seq analysis of T-cells infiltrating vitiligo, primary melanoma and metastasis lesions of patients treated with anti-PD1 immunotherapy. **(A)** Alluvial plots depicting common V(D)J TCR rearrangements between PBMCs, primary melanoma, vitiligo lesions and metastasis (when present) of the same patient. Alluvial flow diagrams are designed to describe common patterns in an evolving network. The division and merging of common TCR sequences can be viewed as multiple streams that flow smoothly throughout different samples of the same patient. **(B)** Productive Simpson clonality of TCR clonotypes sequenced in primary melanoma, vitiligo developed during therapy (blue), metastases, and PBMCs samples. Specimens from the same patient are color-matched. Metastasis samples were collected from different sites: lymph node, spleen and omentum. Simpson clonality score ranges from 0 to 1. Values near 1 represent samples with one or a few predominant rearrangements (monoclonal or oligoclonal samples) dominating the observed repertoire. Clonality values near 0 represent more polyclonal samples. Nonparametric Kruskal-Wallis test was used to analyze the difference in TCR clonality between different sample types (*p-value* = 0.01393). Two-stage linear step-up procedure of Benjamini, Krieger and Yekutieli was used for *post-hoc* analysis (*p value* vitiligo vs metastasis = 0.0139; primary melanoma vs metastasis= 0.0139).

The TCR sequence analysis revealed a differential productive TCR clonality among tissues (peripheral blood: range 0.0081-0.2048; n=10; primary melanoma: range 0.0035-0.2401; n=13; vitiligo tissues: range 0.0053-0.0609; n=10. metastasis: range 0.0078-0.199; n=5) and productive templates as shown in **Figure 5B** (*p* value=0.0139).

Notably, we observed common V(D)J TCR rearrangements between samples derived from the same patients at different time points (**Table 2** and **Figure 5A**). Clonotypes shared by metastasis, surgically removed during the anti-PD-1 therapeutic period, and vitiligo samples are higher compared to the number of clonotypes shared by primary melanoma and vitiligo specimens. Interestingly, we observed the highest number of shared clonotypes between vitiligo and primary melanoma in the two patients achieving iCR (n=21 and n=15; **Table 2** and **Figure 5A**). Furthermore, shared TCR clonotypes have been sequenced in primary melanoma and in metastasis of all the four patients, underlying the possibility that tumor-infiltrating T cells sharing the same specificity, are present in both primary and metastatic sites.

The TCR sequences of PBMC samples has been performed at the time of vitiligo occurrence, and as expected PBMCs showed the lowest Simpson clonality respect to the other tissues. It was also noted that several TCRs sequenced in the PBMCs samples were already present in the primary melanoma or in the metastasis, as shown in **Table 2** and in **Figure 5**.

### 3.4 Immune cell profile of primary melanomas in patients who developed or not vitiligo during immunotherapy and in matched metastases

Flow cytometry analysis showed that frequency of Th17, NK, and CD8-MAIT cells was differently modulated in the blood of patients developing vitiligo compared to those who did not develop it. To investigate whether a correspondence could be seen between circulating immune cell profiles and those present in the tumor tissues, we analyzed by immunohistochemistry and immunofluorescence the primary melanomas of patients who developed vitiligo and primary melanoma of three patients who did not developed vitiligo during therapy, selected from patients listed in **Table 1** (IMM-9, IMM-11, and IMM-12), as a tissue reference to T0 circulating samples. Lymph node metastases from patients VIT_02, VIT_04, and VIT_08 (**Table 2**), who developed vitiligo, were chosen as a reference for on-going therapy reference tissues, in addition to vitiligo skin samples examined in **Figures 3**, **4**.

**Figure 6:**
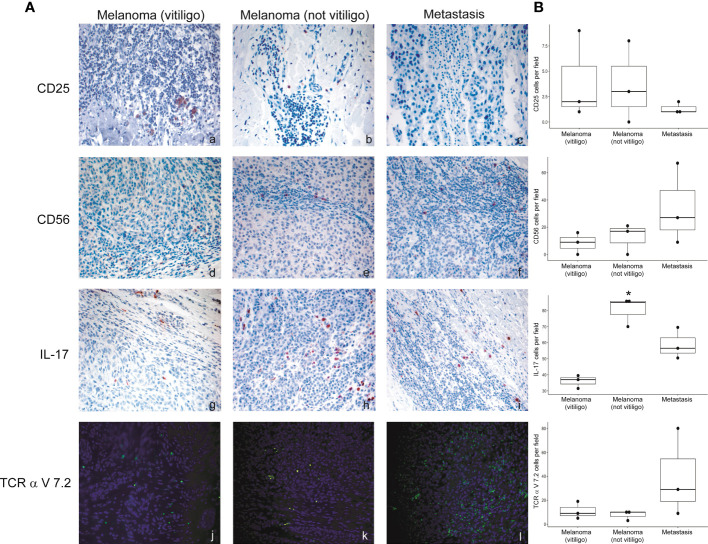
Expression of the immune cell subsets in the primary melanoma and metastases of patients developing or not vitiligo during anti-PD-1 therapy. **(A)** Representative images of immunohistochemistry (panels a to i) and immunofluorescence (l–n) obtained using anti-CD25 (a–c), anti-CD56 (d–f), anti-IL-17A (g–i), and anti-TCR alpha V 7.2 (j–l) in primary melanoma and metastasis from patients who developed (vitiligo) or not vitiligo (not vitiligo) during therapy. A–I 100x magnification, panel L-N 200X magnification. Magnification 200x. **(B)** Quantitative analyses of immunohistochemical staining. The mean value + standard error of the mean (SEM) of the cell count obtained for five different fields is shown, *p<0.05 as assessed by Kruskal-Wallis test.

No significant differences were observed for cells stained with anti-CD56 and anti-TCR V alpha 7.2 antibodies. We detected a trend of lower CD25 positive cells in the lymph nodal metastases in respect to primary melanomas (**Figure 6**). Interestingly, a significative lower frequency of IL-17A positive cells was detected in primary melanomas of patients developing vitiligo during therapy in respect to patients not developing this irAE (**Figure 6**).

## 4 Discussion

Antibodies that block immunological checkpoints can result in long-lasting benefit for patients with many different malignancies. PD-1 is one of the first immunologic checkpoint to be clinically targeted and the anti-PD-1 antibodies Nivolumab and Pembrolizumab have been shown to improve overall survival in a subset of metastatic melanoma patients (30). Identifying patients who are most likely to benefit from PD-1 blockade and why they respond to such a therapy remains an active area of investigation. Since Nivolumab and Pembrolizumab exert their antitumor effects through T-cells, several studies have investigated T lymphocyte populations and correlates immunological changes with patient outcomes (31). Other investigations have focused on T-cell receptor (TCR) repertoire and have proved that PD-1 blockade induces diversification of TCR repertoire status 2 (32, 33) before and after immunotherapy and results in improved prognosis (34, 35).

In this study, we used available blood and tissue samples from melanoma patients who developed vitiligo, a known irAE emerging upon anti-PD-1 therapy, to further understand the immune mechanisms characterizing vitiligo onset and anti-tumoral responses. Vitiligo is an immune-mediated disease involving a complex relationship between immune system and melanocytes physiology (36). A causality of vitiligo is the melanocyte oxidative stress, an initial condition often present in the earliest stages of the disease. The consequence of the oxidative stress is an early activation of the innate immune cells by recognition of damage-associated molecules, which in turn stimulates an adaptive immune response accountable for the anti-melanocyte immunity. Specifically, the release of reactive oxygen species by stressed melanocytes leads to production of several damage-associated molecules recognized by dendritic cells that, as a result, trigger autoreactive T lymphocytes. Impairment of T-reg cell function, as well as reduction in T-reg cell number, further enhance CD8^+^ T cell autoreactivity against melanocytes (36).

We found that vitiligo onset in anti-PD-1 treated patients was characterized by reduction of circulating Th17, CD8-MAIT, and T-reg cells. Th17 lymphocytes are a subset of CD4^+^ T cells implicated in the pathogenesis of various autoimmune diseases. Th17 cells secrete several immune modulating molecules including IL-17 and IL-22 (37). Previous data reported a significant correlation between Th17 cells and IL-17 with spontaneous vitiligo and indicated an involvement of Th17 cells in vitiligo progression and severity (38). Th17 cells and IL-17 expression are higher at the leading edge of a vitiligo lesion compared to a non-lesional skin (39). Consistently, in melanoma patients under immunotherapy, we found that Th17 cells are reduced in the blood of subjects developing vitiligo compared to those not developing the irAE and that IL-17A-expressing cells are enriched in skin biopsy of immunotherapy-mediated vitiligo compared to the spontaneous disease. These results suggest that Th17 cells are involved in pathogenetic mechanisms of both immune therapy-induced and spontaneous vitiligo. Moreover, the higher amount of IL-17A positive cells in the anti-PD-1 therapy-dependent vitiligo may indicate that Th17 cells in the skin lesion could be a distinctive feature of vitiligo as an irAE. In addition, the reduction of Th17 cells in the blood of these patients could be related to cell migration into the skin lesion. The role of Th17 cells in vitiligo pathogenesis is still unclear. A mechanism could involve the cytokine IL-17A, which may act as a chemoattractant for cytotoxic CD8^+^ T cells and recruit melanocyte-specific T lymphocytes to the skin (38). Additionally, IL-17A has been shown to antagonize melanogenesis and promote melanocyte apoptosis, two mechanisms causing depigmentation in vitiligo (38).

Interestingly, our analysis of primary melanomas from patients who developed or not vitiligo under anti-PD-1 therapy showed a higher number of IL-17A-expressing cells in the tumor samples of patients not developing vitiligo. Since all the patients examined were responding ones, expression of IL-17A does not have here any prognostic value. However, this data could indicate that vitiligo development mainly derived from the individual response to anti-PD-1 therapy and not from primary melanoma immune features.

Another cell population able to release IL-17 is represented by MAIT cells, whose physiological role is the defense of mucosa from bacterial and mycobacterial infections (40). In our study, we detected a lower frequency of CD8-MAIT cells in the blood of melanoma patients with the onset of vitiligo. Given their ability to produce IL-17, we hypothesize common mechanisms of action with Th17 cells. Moreover, other cell types could express IL-17A, such as NK cells (41) or γ/δ T cells (42) further sustaining a role for innate immunity in vitiligo pathogenesis. However, no significant differences were found in our cohort of patients regarding the amount of circulating NK and γ/δ T cells between patients developing or not vitiligo during anti-PD-1 therapy.

We previously observed that a lower number of T-reg cells before therapy initiation was present in the blood of patients, positive responders on anti-PD-1 therapy in respect to not responders (14). In that case, we did not observe a reduction of circulating T-reg throughout the treatment. Here, we found a frequency reduction of circulating T-reg cells in patients developing vitiligo during therapy. Therefore, since not all the responding patients develop vitiligo, our data indicate that the reduction of circulating T-reg cells is specifically related to vitiligo occurrence, as previously reported (36). Of note, the trend of lower CD25 positive cells we observed in the metastatic tissues of the four patients who developed vitiligo, in respect to primary melanomas, could also be interpreted as a reduction of T-reg cells connected with vitiligo development.

Interestingly, a common mechanism shared among all patient groups was the reduction of circulating NK CD56^bright^ cells during treatment, a phenomenon possibly related to the positive response to therapy. Nevertheless, comparison of data from complete and partial responders with the ones of progressive disease patients did not highlight a significant difference in blood frequency of NK CD56^bright^ (our unpublished data). The cytotoxic activity of NK CD56^bright^ cells is lower than that of the NK CD56^dim^ cells, thus a reduction in the number of NK CD56^bright^ cells during treatment could represent their switch to a more effective cell type, potentially reflecting a positive response to immunotherapy (43). Moreover, we observed that PD-1 expression, reduced in most cell subset after anti-PD-1 treatment, is significantly increased on NK CD56^brigh^ cells from subjects developing vitiligo. This result indicates that NK CD56^brigh^ cells are not responsive to anti-PD-1 therapy and suggests that PD-1 expression on NK CD56^brigh^ cells could be upregulated by a compensatory mechanism triggered by therapy and could be related to response to therapy and/or vitiligo development. Consistently, it has been demonstrated a positive correlation between PD-1 expression on NK CD56^brigh^ cells and overall survival of patients with lung cancer undergoing immunotherapy (44). However, a higher number of patients should be considered to clarify this point.

In order to understand whether immunotherapy would induce reactivation of T cell clones already present in the primary melanoma and whether T cell clones were directed against antigens common to melanoma cells and normal melanocytes, we investigated, by TCR sequencing, the T cell clonality in patient’s matched specimens from primary melanoma and vitiligo lesion developed after anti-PD-1 therapy. In fact, a previous study identified one common TCR clone in the primary tumor and in the concomitantly developed vitiligo lesion in a patient affected by cutaneous melanoma (10). We found that although in vitiligo lesions TCR sequences were generally different from those in the primary melanoma, shared clonotypes could be identified between the vitiligo tissue and primary or metastasis site, especially for those patients achieving a iCR upon anti-PD1 therapy. Moreover, in all evaluated patients, the number of shared TCR clonotypes between vitiligo and metastatic sites was superior compared to that observed in the primary tumor, underlying that the T cells immunoreacting against normal melanocytes are also able to selectively infiltrate the metastatic sites.

These data are also indicating that the immunotherapy-stimulated T cell response against normal melanocytes, which is involved in vitiligo onset, is not mediated by the reactivation of specific T cell clones infiltrating the primary melanoma but may be elicited *de novo* by T cell clones targeting metastatic tissues. In fact, all the patients we examined had primary melanoma surgically removed before immunotherapy initiation, whereas they still presented distal metastasis. Interestingly, the two patients achieving complete response under anti-PD-1 therapy were also the unique two patients showing high amount of T cell clones common to primary melanoma and vitiligo. We suppose that these T cell clones are directed against tumor-associated antigens, which are mainly found in tumor cells, but can also be expressed by normal melanocytes. In fact, melanoma tumor antigens could be grouped in two types: antigens that are specifically expressed by tumor cells, also known as neoantigens or tumor antigens, and tumor-associated antigens, shared among tumor cells and normal melanocytes (45). Although previous studies claimed that tumor-associated antigens do not induce an effective cytotoxic T cell response and tumor-specific CD4^+^ T lymphocytes (45), our present data suggest that tumor-associated antigens have a predominant role in advanced melanoma. In line with the concept of “beneficial autoimmunity” (13), the presence of shared TCRs able to recognize tumor-associated antigens in matched vitiligo and metastatic lesions of positive responders, strongly suggests the active role of these T cell clones in fighting advanced forms of tumor. In addition, the assessment of specific T cell clones against tumor-associated antigen in primary tumors of complete responders further supports this concept, highlighting the beneficial contribution that these specific cells may have in driving the complete resolution of the tumoral lesions.

Finally, we found that common TCR clones were present in the primary melanomas and in the metastasis of patients experiencing tumor relapse during immunotherapy, as well as in their PBMCs at the time of vitiligo onset. Similar data have been already reported for one patient affected by uveal melanoma (11). In this context, Riaz et al. previously demonstrated that tumor cell mutations, potentially recognized by T cells in the pre-therapy melanoma samples, were still detectable in on-therapy tumor tissues, and that a high proportion of mutations detectable during immunotherapy was associated with treatment resistance (46). Actually, mutation burden decreases with successful checkpoint blockade therapy in patients with melanoma, suggesting that immune cell selection against mutant neoepitopes may be a critical mechanism of response to therapy (46).

This study is limited by the sample size, in part due to the low frequency of vitiligo irAE (15%) in melanoma patients who were treated with checkpoint inhibitor therapy (9). In fact, we enrolled more than 40 patients to select those analyzed in this study. However, this pilot study gives indications for future validation with a larger sample size.

Even considering the bias of the small numbers, our results still represent consequent frames showing the dynamic development of tumor immune responses induced by the anti-PD-1 immunotherapy, also causing the irAE vitiligo in some patients. Considering the prognostic significance of vitiligo onset during immunotherapy, our analysis of the immune profile of blood and tumoral tissues of melanoma patients sheds light on immunological mechanisms in melanoma setting. The cell mechanisms demonstrated here will contribute to the identification of potential biomarkers of response, as well as novel therapeutic targets.

## Data availability statement

The datasets presented in this study can be found in online repositories. The names of the repository/repositories and accession number(s) can be found below: GSE229557 (GEO).

## Ethics statement

The studies involving humans were approved by Istituto Dermopatico dell’Immacolata (IDI)-IRCCS. The studies were conducted in accordance with the local legislation and institutional requirements. The participants provided their written informed consent to participate in this study. The study was conducted in accordance with the local legislation and institutional requirements.

## Author contributions

Conceptualization, MC, CQ, EV, and CF; methodology, MC, AC, MG, SR, and DL; investigation, MC, AC, MG, SR, DS, DL, and CR; data curation, MC, AC, MG, DS, DL, and DP; writing original draft preparation, MC, MG, EV, CQ, and CF; writing review and editing, MC, AC, MG, DP, CQ, EV, and CF; supervision, CQ, EV, and CF; funding acquisition, EV, CQ, CF. All authors contributed to the article and approved the submitted version.
