# Supplementary material for: Insight into immune profile associated with vitiligo onset and anti-tumoral response in melanoma patients receiving anti-PD-1 immunotherapy
Source: Front Immunol. 2023 Aug 23;14:1197630. doi: 10.3389/fimmu.2023.1197630 (PMC10482109; doi:10.3389/fimmu.2023.1197630)
Supplement: Supplementary file 2 [file DataSheet_2.docx]

Supplementary Material

Insight into immune profile associated to vitiligo onset and anti-tumoral response in melanoma patients receiving anti-PD-1 immunotherapy

**
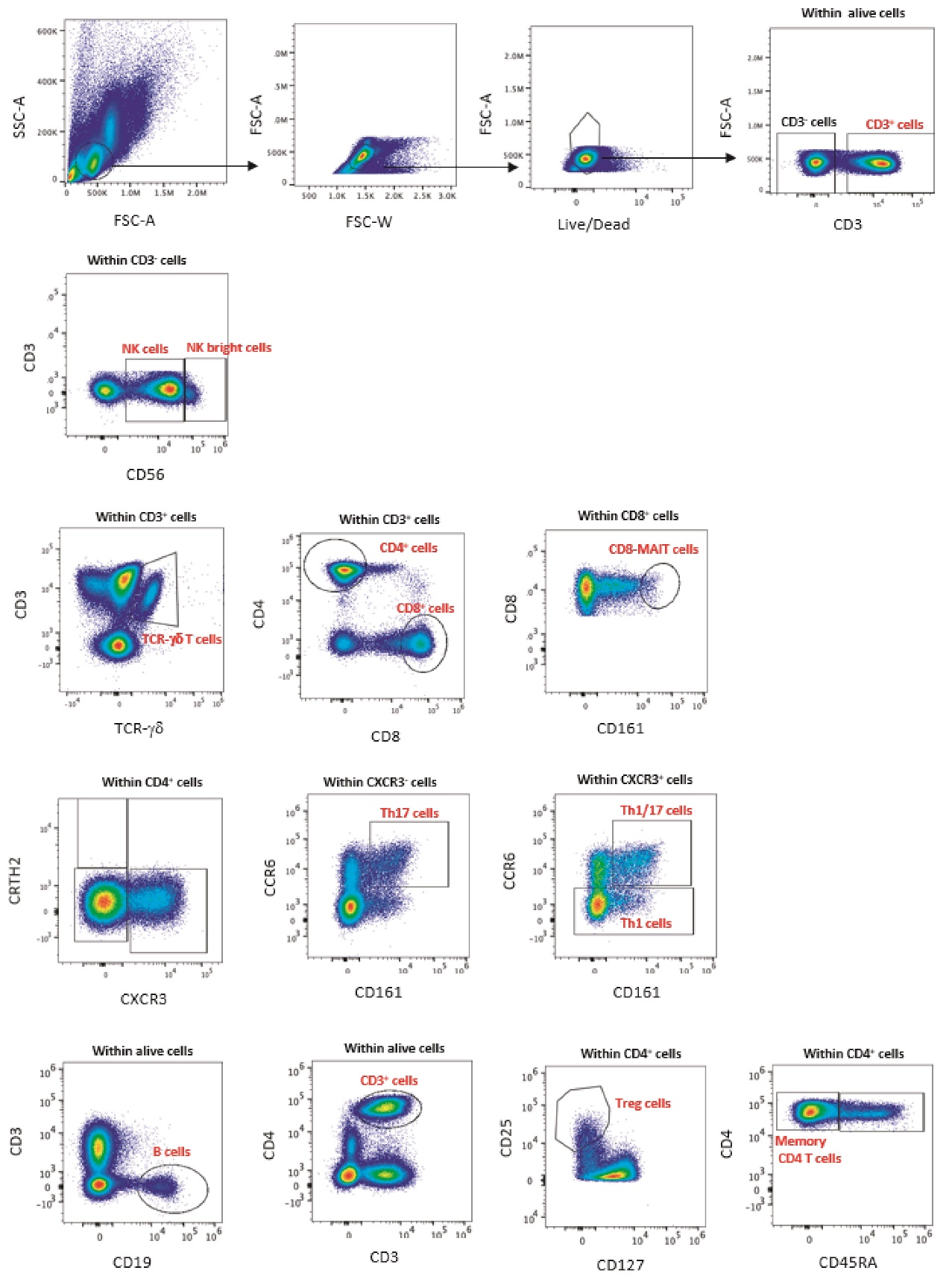
**

**Supplementary Figure 1. Gating strategy for the discrimination of immune cell types.** Human PBMCs purified from peripheral blood of healthy donors were labelled with specific antibodies conjugated with a fluorochrome. The plots show the data of a representative of more experiments.


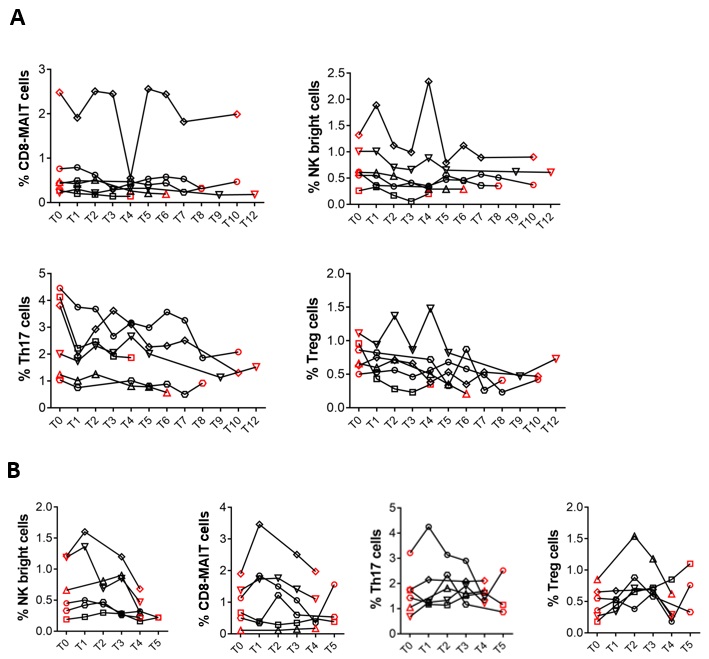


**Supplementary Figure 2. Time course of immune cell frequencies modulated in the blood of melanoma patients developing or not vitiligo during anti-PD-1 therapy.** Blood frequencies of CD8-MAIT, B, NK bright, Th17 and Treg cells were analyzed by flow cytometry in PBMCs of melanoma patients before therapy (T0), and at the different time points reported in the Materials and methods in (A) patients developing vitiligo, and (B) patients not developing vitiligo.


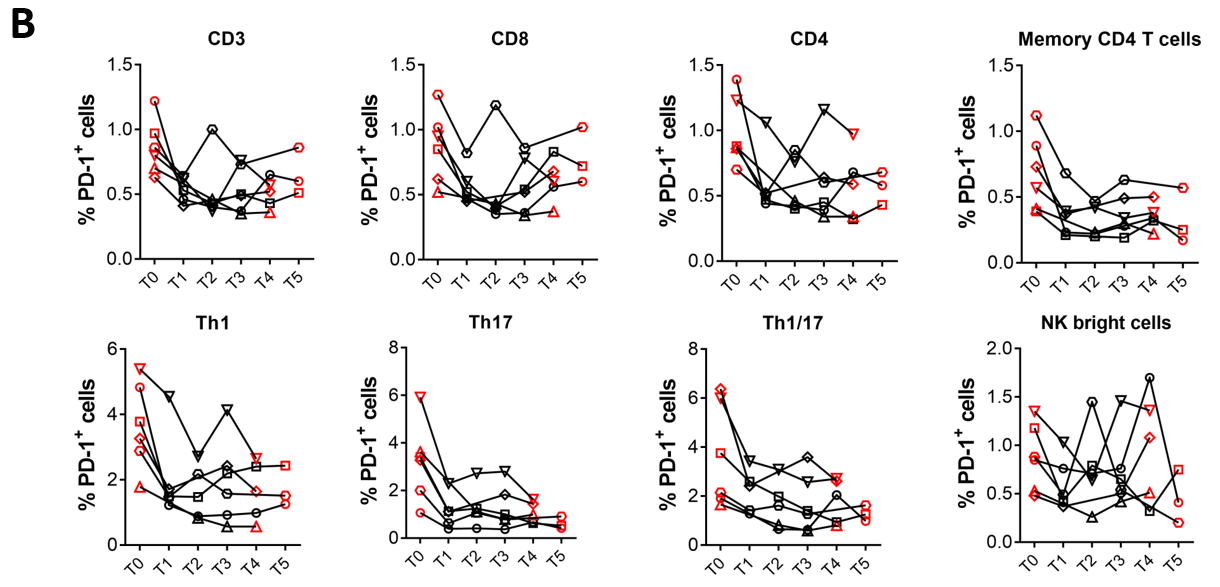


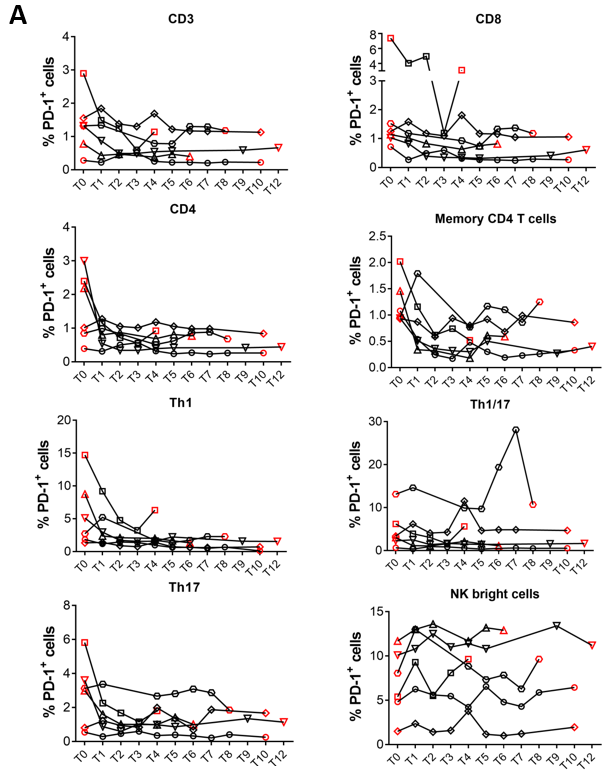


**Supplementary Figure 3. Time course of PD-1 expression modulated in the blood of melanoma patients developing or not vitiligo during anti-PD-1 therapy.** Percentage of PD-1 positive cells  within CD3+, CD8+, CD4+, memory CD4, Th1, Th1/17, Th17, NK bright cells in PBMCs of melanoma patients before therapy (T0), and at the different time points reported in the Materials and methods in (A) patients developing vitiligo, and (B) patients not developing vitiligo.

**Supplementary Table I.** For each sample for which TCR sequencing was performed, metrics such as total templates, productive templates, productive rearrangements, Simpson clonality are reported.

**Supplementary Table II.** Nucleotide sequence of CDR3 unique region of shared TCR-β clonotypes and the productive frequency.
